# Case Report of a Dermatologic Reaction to Wound Closure Strips and Liquid Adhesive

**DOI:** 10.21980/J8.52256

**Published:** 2025-10-31

**Authors:** Amal Asghar, Trevor Smith, Matthew Underwood, Tommy Y Kim

**Affiliations:** * HCA Healthcare, Riverside Community Hospital, Department of Emergency Medicine, Riverside, California

## Abstract

Type IV hypersensitivity reactions are delayed, cell-mediated reactions between T cells and antigens that normally occur usually within 48 to 72 hours after exposure to an antigen. This is a case of a 51-year-old male status post left Achilles tendon repair with findings consistent with a type IV hypersensitivity reaction on post-operative day 12. The patient’s examination revealed blistered and macerated skin underneath the region of the wound closure strips and liquid skin adhesive. Wound care and debridement were initiated on post-operative day 14 by a wound care specialist. After six weeks of weekly wound care with debridement, the wound ultimately healed. Clinicians should be aware of the ability for any patient to develop a delayed type IV hypersensitivity reaction to treatment regimens, including dermatologic wound closure strips and liquid skin adhesives.

**Topics:**

Type IV hypersensitivity reaction, wound closure strips, liquid skin adhesive.

**Figure:**
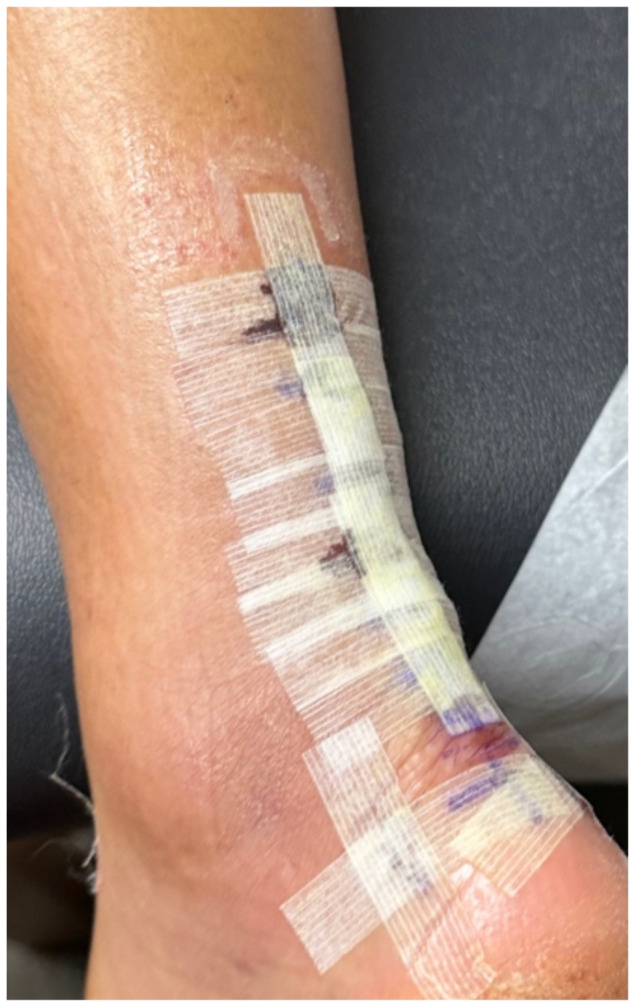


**Figure:**
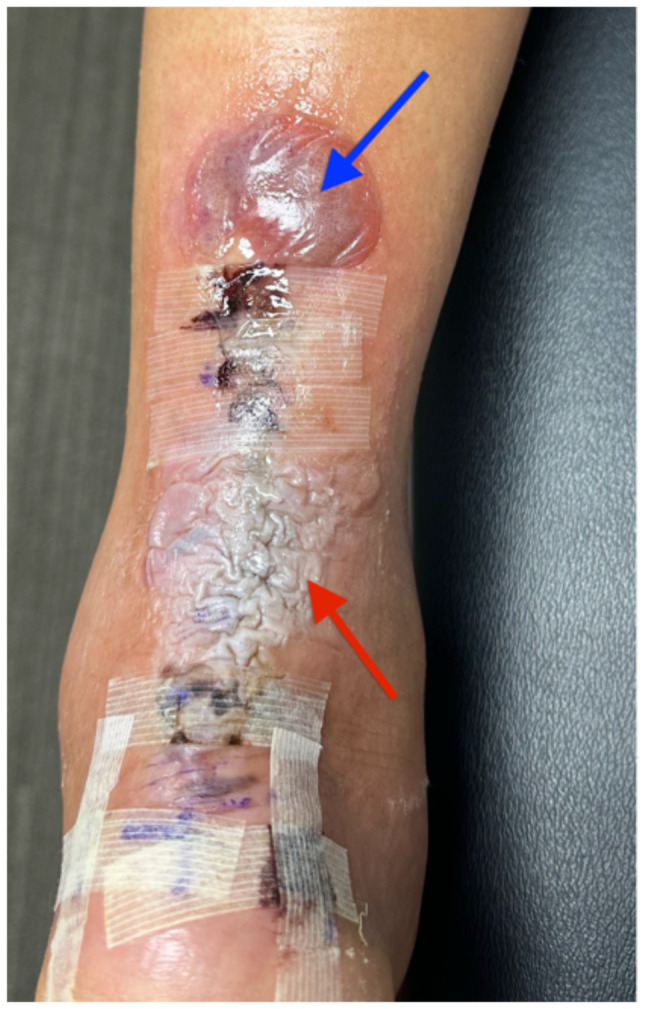


**Figure:**
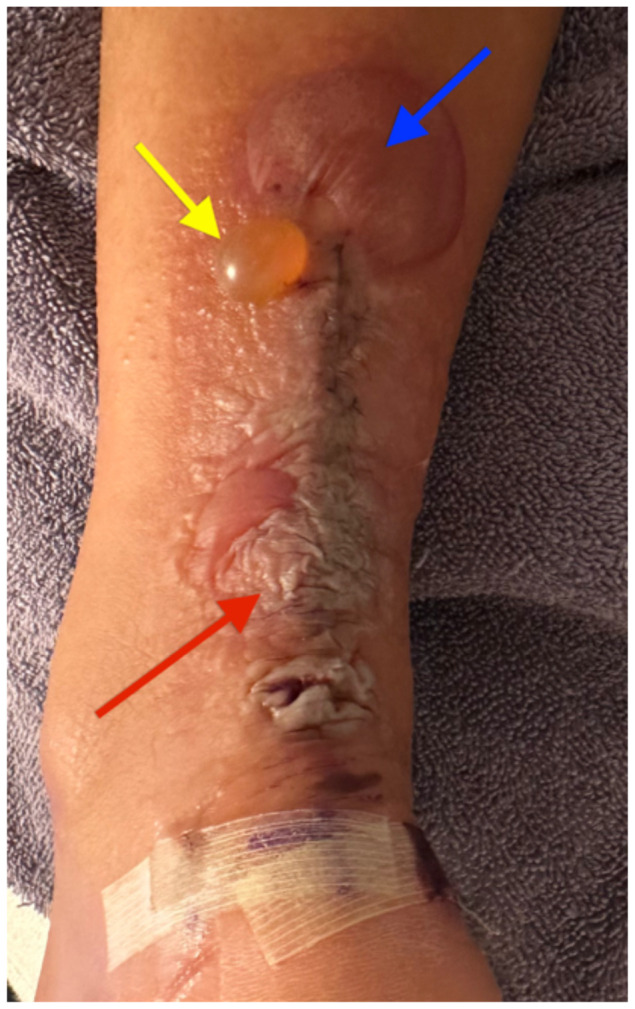


**Figure:**
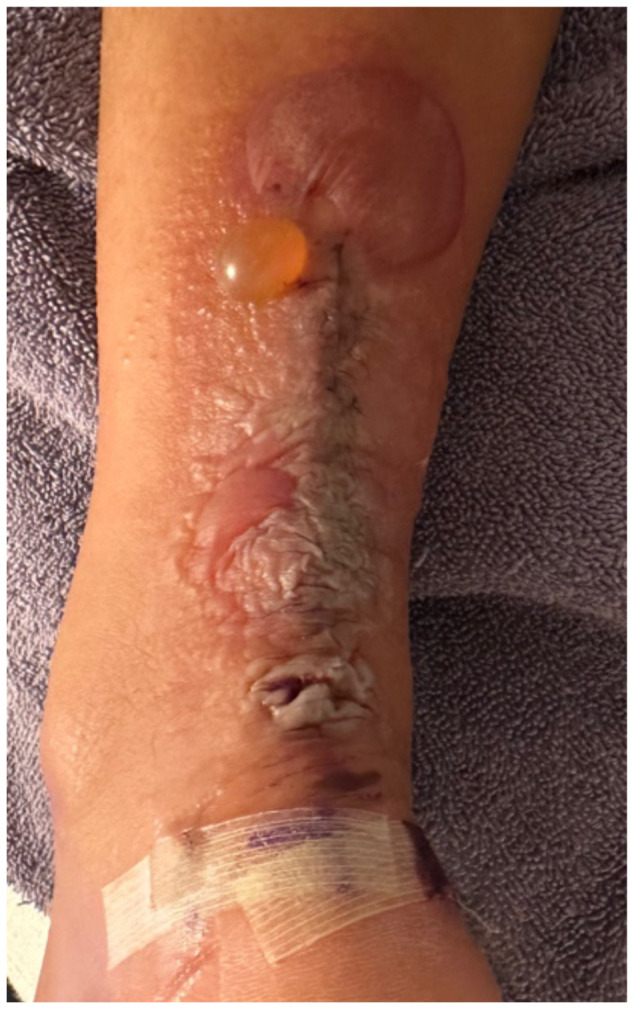


**Figure:**
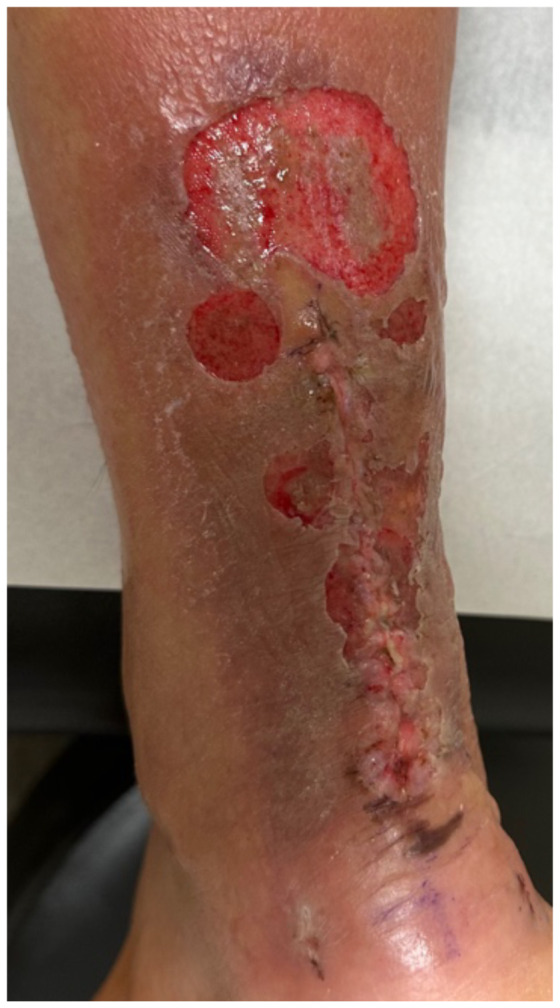


**Figure:**
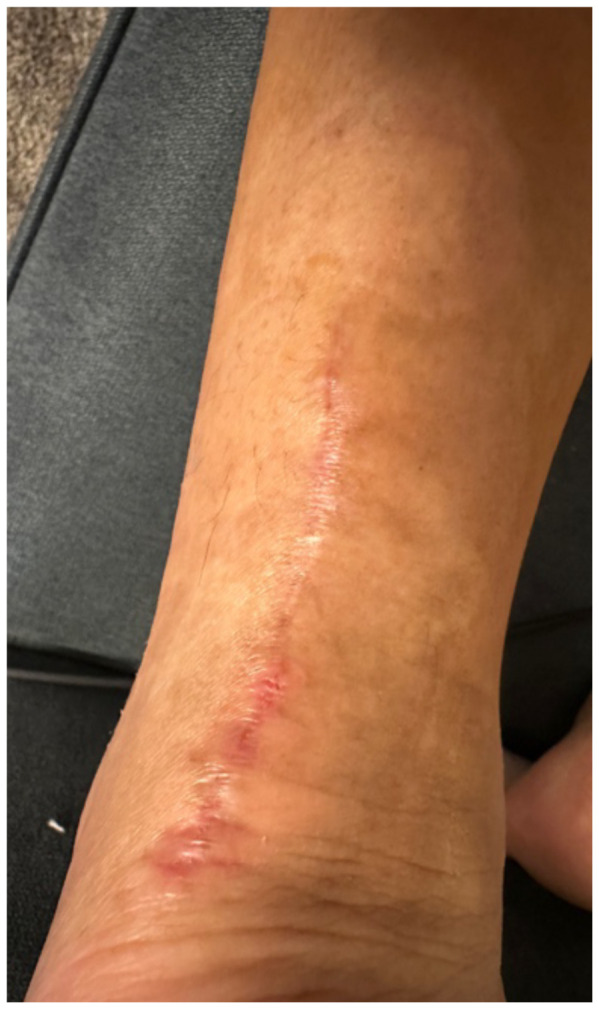


## Brief introduction

Type IV hypersensitivity reactions are delayed, cell-mediated reactions between T cells and antigens. These reactions are delayed due to the late onset of symptoms that normally occur more than 12 hours after contact; usually within 48 to 72 hours. At first contact, the antigen induces an immune response activating nearby leukocytes. These macrophages and monocytes present the antigens to T cells, which release cytokines that ultimately cause inflammation and tissue damage.^1^ What starts as a beneficial T lymphocyte-mediated response to a foreign antigen develops into a harmful, destructive process involving repeat exposure of the antigen resulting in further inflammatory damage.^2^ Allergic contact dermatitis is an example of disease caused by type IV hypersensitivity reaction. Common substances that may induce dermatitis include gloves, lotions, nail products, poison oak, and nickel. While the average time stated in the literature from exposure to dermatitis symptoms is 48 hours, few cases have shown dermatitis symptoms developing past 72 hours.^3^ Due to the high prevalence of allergic contact dermatitis, it is worth exploring cases in which patients present past 72 hours with dermal symptoms.

## Presenting concerns and clinical findings

This is a 51-year-old male, who ruptured his left Achilles tendon while playing volleyball, underwent surgical repair with deep wound closure with 5-0 synthetic absorbable monofilament sutures, and superficial closure with wound closure strips and liquid medical adhesive: ingredients included gum mastic, styrax, methyl salicylate, ethanol, and acetone. On the initial day 6 post-operative visit, the wound appeared to be healing well. On post-operative day 11, the patient began to develop discomfort with itching and pain.

## Significant findings

The patient removed the splint, and the wound were notable for erythematous bullae (blue arrow), blisters (yellow arrow), and skin maceration (red arrow) in the distribution under the wound closure strips. Of note, there was no surrounding erythema with poorly defined borders.

## Patient course

The patient followed up with orthopedics on post-operative day 13 as more blisters formed under the wound closure strips, and at that time, cultures were obtained and the patient was started on trimethoprim/sulfamethoxazole. The patient was immediately referred to a wound specialist. Wound care and debridement were initiated on post-operative day 14 by the wound care specialist. Patient self-medicated with Cetirizine and triamcinolone cream around the outer wound margins. The wound culture returned negative, and the trimethoprim/sulfamethoxazole was stopped. After six weeks of weekly wound care with debridement, the wound ultimately healed.

## Discussion

This case described a patient who likely developed a delayed type IV hypersensitivity reaction to wound closure strips and liquid skin adhesives. Though most delayed type IV hypersensitivity reactions develop symptoms within 72 hours, the patient did not develop symptoms until post-op day 11. It is worth exploring whether the patient truly had an allergic reaction or another pathology with a similar presentation. Though our patient had characteristic pruritus and erythematous bullae characteristic of an allergic skin reaction, the timing could suggest an infectious pathology.

Another potential confounding variable is if our patient was allergic to the wound closure strips and liquid skin adhesives or to the absorbable sutures used to close the wound. The distribution of the bullae and blisters consistently covers the skin directly underneath the wound closure strips instead of just the skin surrounding the puncture sites of the sutures, more characteristic of an allergic reaction to the wound closure strips and liquid skin adhesives. Fortunately, the patient took clear pictures detailing the incidence and recovery of his reaction allowing for a more objective analysis. The pictures are sensitive enough for inference, possibly diagnostic of an allergic skin reaction, but not quite as specific for allergic reaction versus bacterial cellulitis. Although the negative culture results do point more towards an allergic reaction compared to inference. The lack of diagnostic certainty calls for physicians to always keep other disease processes on their differential such as allergic contact dermatitis, irritant contact dermatitis, cellulitis, necrotizing soft tissue infection, statis dermatitis, lipodermatosclerosis, lymphedema, and other categories of disease.^3^

Although our patient did not have skin patch allergy testing done to determine the culprit allergen, other case reports have shown sensitivity to wound closure strips and liquid skin adhesives.^4^,^5^ Clinicians should be aware of the ability for any patient to develop allergic reactions to treatment regimens, including dermatologic wound closure strips and skin adhesives. Unlike the limitations described in Mestach et al related to the variance of acrylic composition, the composition of materials that make up wound closure strips is consistent.^5^ Patients are more likely to determine which ingredient in the composition of wound closure strips is the culprit of the reaction. As new products and techniques of wound closure are developed, it remains important to be aware of the side effect profile of the materials used in these products. For example, topical skin adhesives containing 2-octyl cyanoacrylate is preferred in wound closure due to its strength and low side effect profile. It is more costly than other liquid skin adhesives, which is why clinicians may choose to use wound closure strips and liquid skin adhesives.^6^ There are multiple studies showing allergic contact dermatitis to liquid skin adhesives alone.^3^,^7^ In such cases, clinicians must determine whether it is the wound closure strips or the liquid tissue adhesive that is causing the skin reaction.

The patient had a delayed type IV hypersensitivity reaction based on the appearance and timing of his clinical features. The key takeaway from this report is that dermatologic reactions often require a broad differential and a close look at the materials for potential causes. Physicians should remember that interventions and treatments carry their own set of risks and benefits that patients assume as they seek medical care. Among these are dermatologic reactions to skin adhesives. Future studies may be focused on providing patients with information at discharge with potential skin reactions to adhesives and appropriate return precautions if they see these signs. Screening methods before surgery may also be of benefit.

## Disclaimer Statement

This research was supported (in whole or in part) by HCA Healthcare and/or and HCA Healthcare affiliated entity. The views expressed in this publication represents those of the author(s) and do not necessarily represent the official views of HCA Healthcare or any of its affiliated entities.

## Supplementary Information














