# Supplementary figures and images for: Case Report of a Dermatologic Reaction to Wound Closure Strips and Liquid Adhesive
Source: J Educ Teach Emerg Med. 2025 Oct 31;10(4):V5–7. doi: 10.21980/J8.52256 (PMC12594465; doi:10.21980/J8.52256)

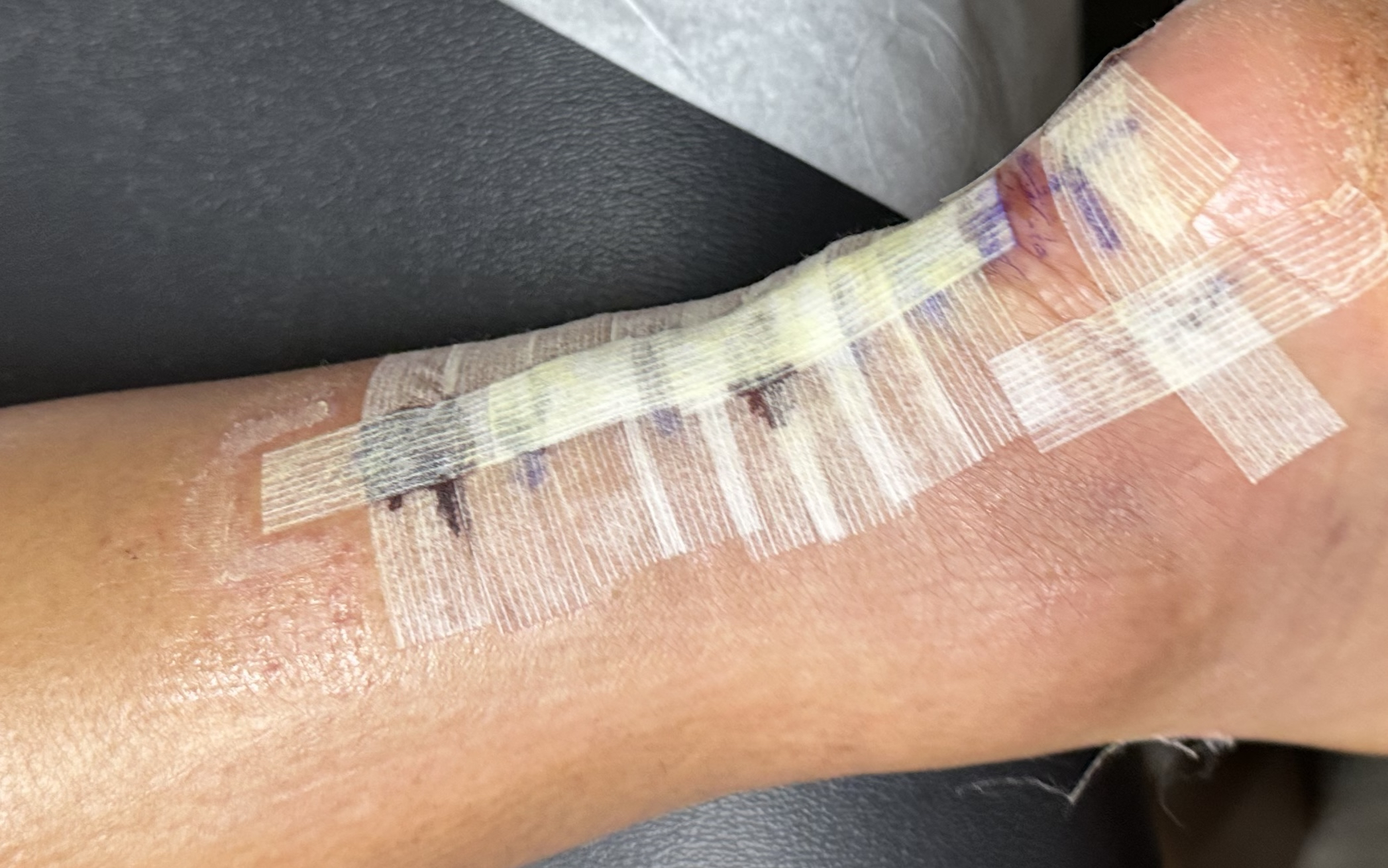

Supplement: Supplementary file 1 [file 10-4-V5-Supp1.jpg]

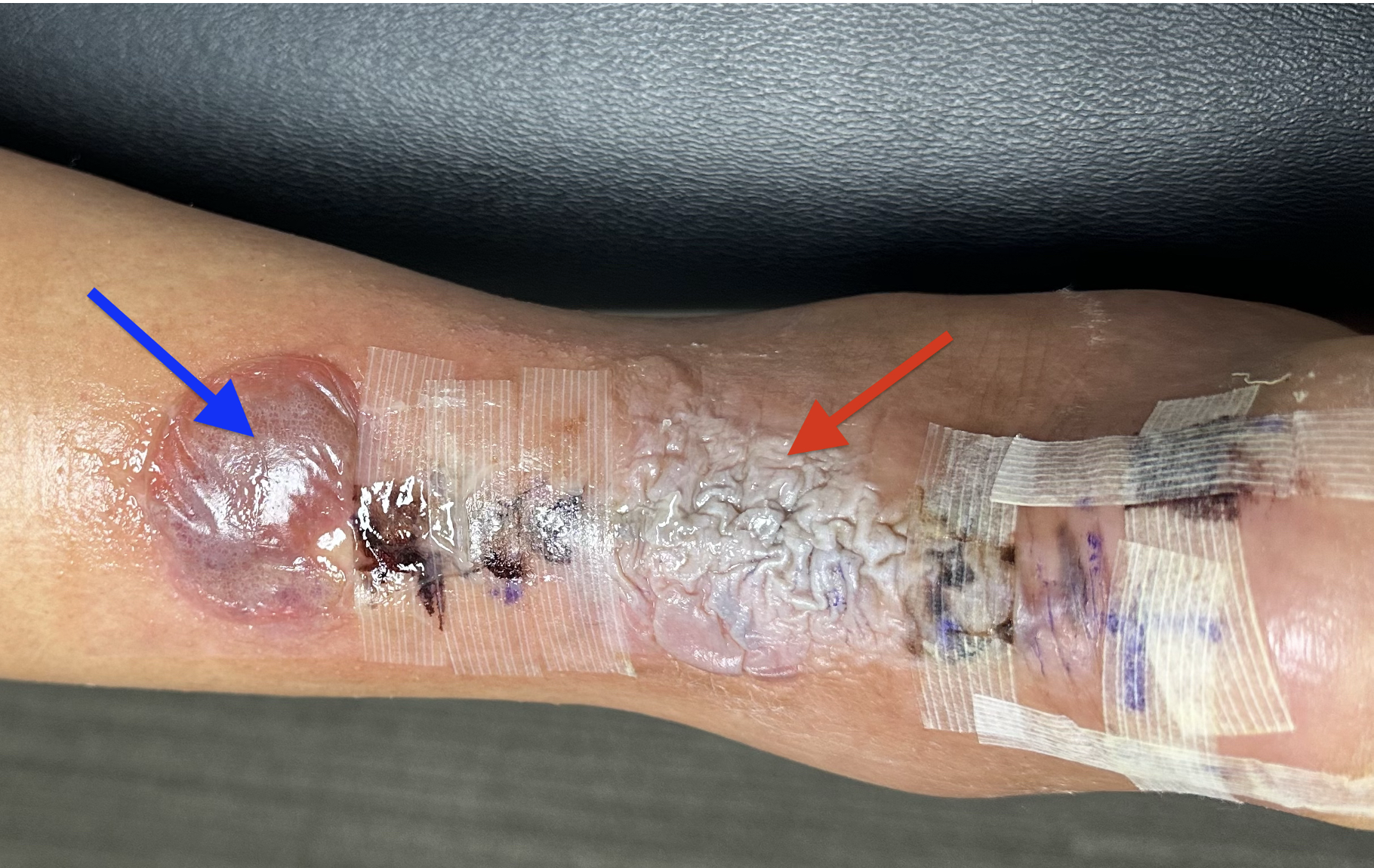

Supplement: Supplementary file 2 [file 10-4-V5-Supp2.png]

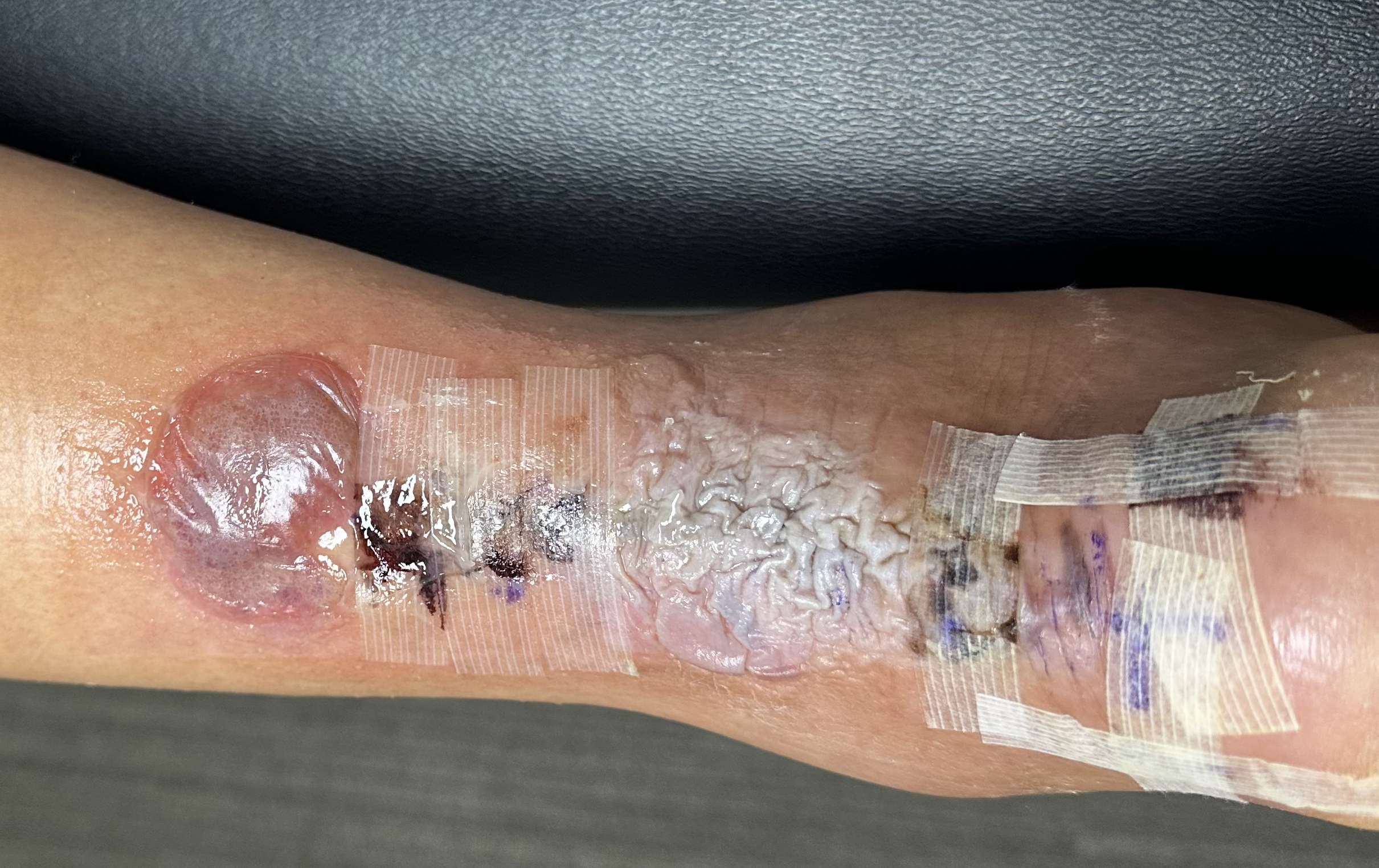

Supplement: Supplementary file 3 [file 10-4-V5-Supp3.jpg]

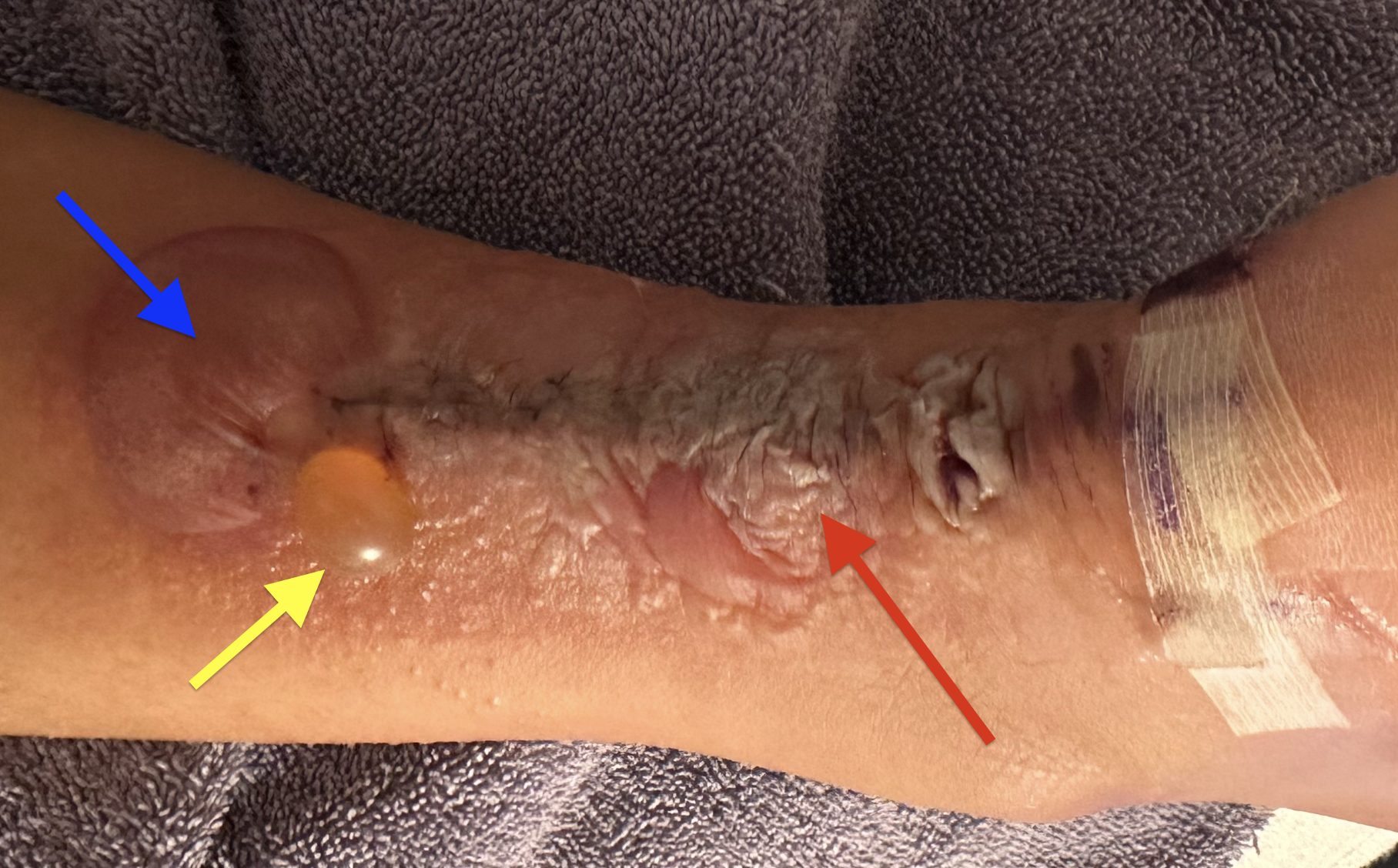

Supplement: Supplementary file 4 [file 10-4-V5-Supp4.png]

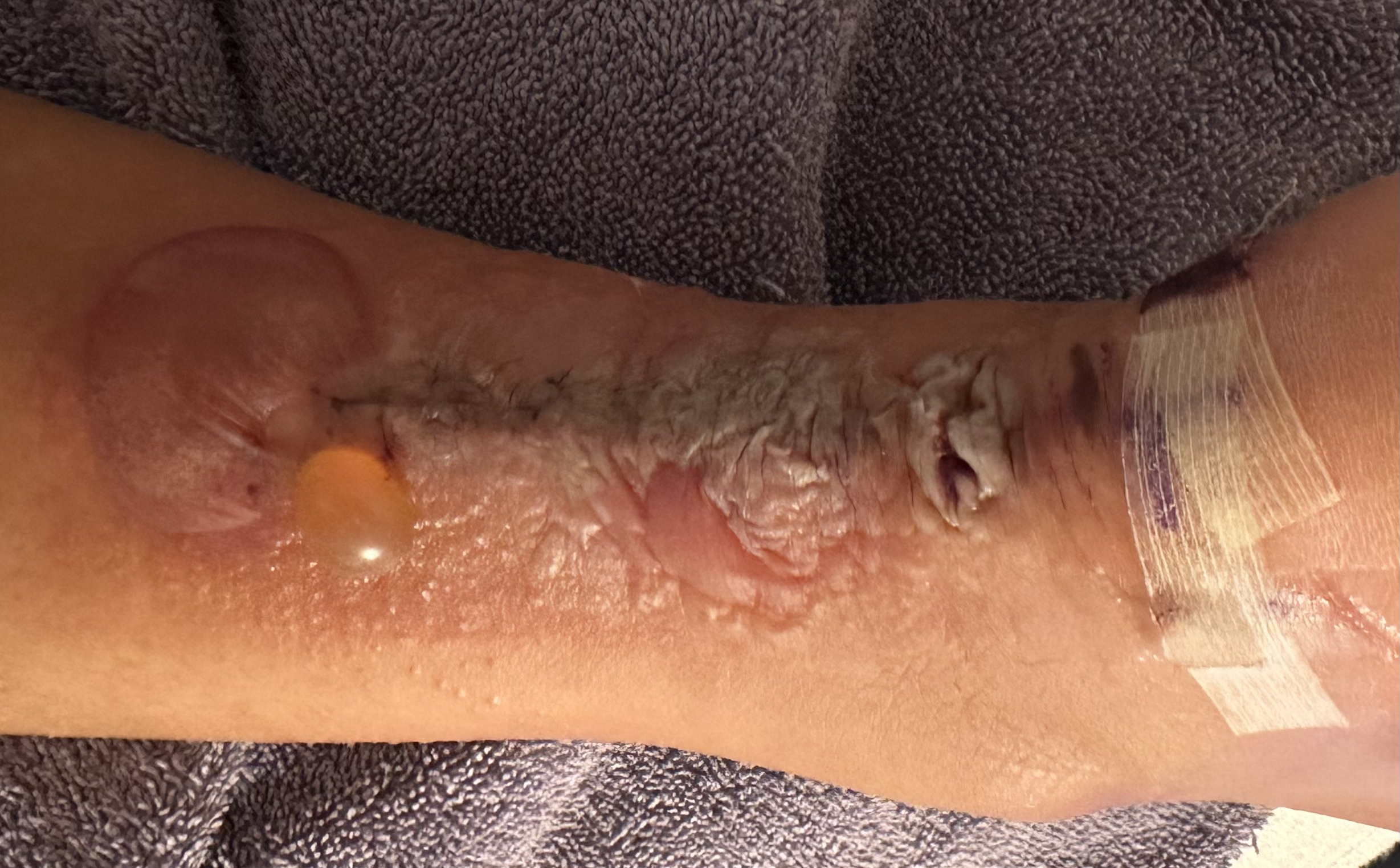

Supplement: Supplementary file 5 [file 10-4-V5-Supp5.jpg]

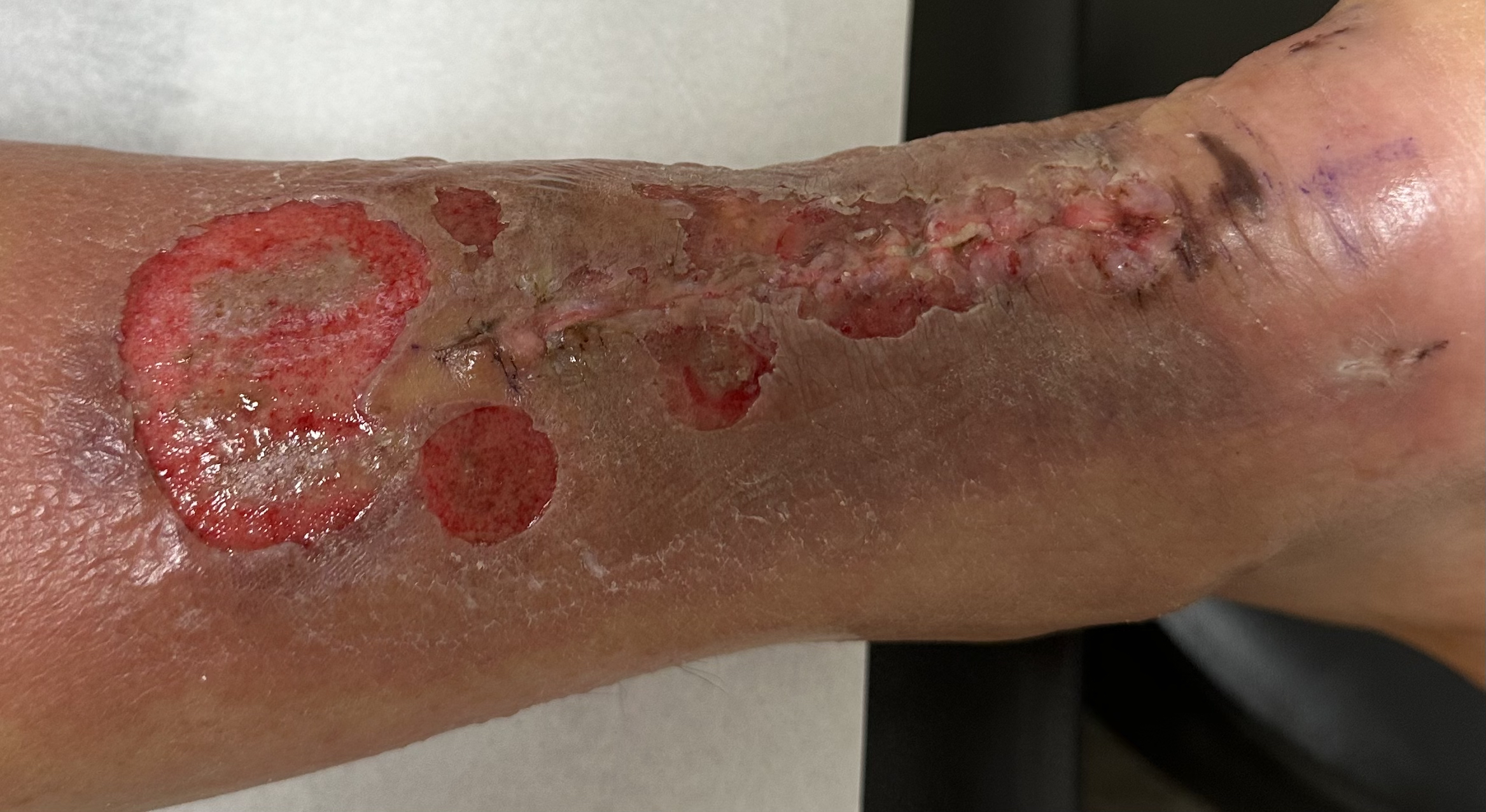

Supplement: Supplementary file 6 [file 10-4-V5-Supp6.jpg]

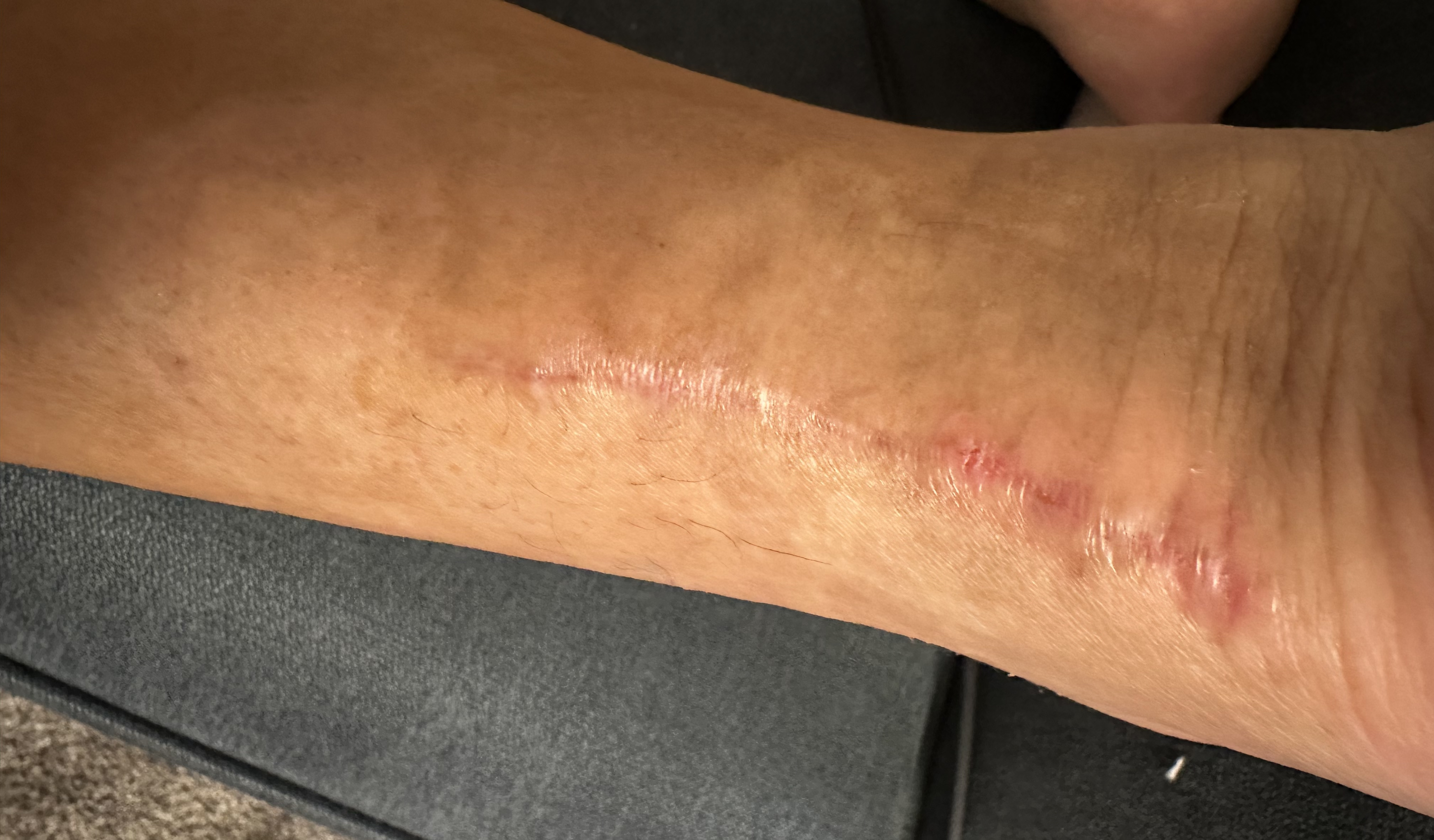

Supplement: Supplementary file 7 [file 10-4-V5-Supp7.jpg]
